# One Health clinic challenges and evolution: increasing access to care for people and pets in a rural community in Northern California

**DOI:** 10.3389/fvets.2025.1599422

**Published:** 2025-06-23

**Authors:** Kristin Jankowski, Kimberly Aguirre Siliezar, Jeannie A. Knuchell, Adrian Duenas-Ramirez, Jennifer J. Edwards, Jonathan D. Dear

**Affiliations:** ^1^One Health Institute, University of California Davis School of Veterinary Medicine, Davis, CA, United States; ^2^University of California Davis School of Veterinary Medicine, Davis, CA, United States; ^3^Department of Medicine and Epidemiology, School of Veterinary Medicine, University of California, Davis, Davis, CA, United States; ^4^University of California Davis College of Biological Sciences, Davis, CA, United States; ^5^Betty Irene Moore School of Nursing, University of California, Davis, Davis, CA, United States; ^6^Department of Medicine and Epidemiology, School of Veterinary Medicine, University of California, Davis, Davis, CA, United States

**Keywords:** One Health clinic, student-run free clinic, spectrum of care, contextualized care, access to care, interprofessional education, rural, underserved

## Abstract

A student-run, free One Health clinic (OHC) improves access to care for people and pets while providing increased training opportunities for interprofessional students in the areas of spectrum of care, contextualized care, cultural humility, ethical community engagement, and relationship-centered communication when clinical instruction is provided. The coordination and implementation of a community-based student-run free clinic (SRFC) that is also an OHC is complex. Programmatic challenges can include coordination with the leaders of multiple training programs, seasonal variation of student and clinical instructor schedules, and the need to balance student experiential learning with positive client and patient outcomes. Internal evaluations of the clinic's scope of care, patient and provider safety, and student preparedness has led to the development of policies and procedures that consider both student training and the client-patient experience. Widening the OHC provider and student partnership to include human nursing was a novel and effective method to enhance care for the bonded family and create opportunities for interprofessional education (IPE) for students from multiple training programs at a single clinical site.

## 1 Introduction

This article describes the challenges and evolution of a student-run, free One Health clinic (OHC) created in partnership with community members of a small, predominantly agricultural community of ~1,000 residents in Northern California. One Health clinics offer a wide range of learning opportunities to students that enhance curricular learning (1, 2). To our current knowledge, the clinic we describe is the only student-run, free OHC in the United States. Student-run free clinics (SRFCs) exist all over the world and vary in scope, services provided, and level of oversight. The common mission of SFRCs is to offer clinical health services to communities facing barriers to care and provide educational opportunities for health professional students (3).

SRFCs provide essential health services and are frequently supported by healthcare professional schools, but there are potential ethical dilemmas associated with SRFCs if proper student training and oversight are not provided (4–6). A balance between student autonomy and faculty involvement during student-led initiatives is optimal, and as our OHC has grown in scope and size, volunteer training has evolved to achieve this balance.

Consistent with the most frequently reported barriers to veterinary care nationwide (7) the community members our clinic serves face financial, language and transportation barriers in seeking care for their pets. According to the World Population Review, more than 25% of the community have an income below 100% of the federal poverty level and 55.44% of community residents speak only English, while 44.56% speak Spanish (8). While the nearest veterinary hospital is only 10 miles away, there is limited public transportation in this region, pets have restricted access on these vehicles (9) and costs of gas for private vehicles can be prohibitive. The OHC, in partnership with the community, serves an essential role in addressing these barriers by providing free, contextualized, culturally sensitive veterinary and human health care while providing interprofessional education for undergraduate and professional students (10).

### 1.1 Context—History and challenges

In response to a lack of geographically accessible human healthcare, community members contacted a university supported student-run human medicine clinic in a larger nearby city about providing care in this rural area. The new medical clinic began seeing patients in 2012, staffed by medical and nurse practitioner students and supervised by licensed physician volunteers. In 2013, the clinic was transformed into a One Health center after community members expressed a lack of accessible veterinary care in their town (11).

### 1.2 Veterinary-human partnership

The veterinary clinic was first held in the winter of 2013 in a parking lot adjacent to the human health clinic and led by students and faculty from the veterinary school. Since that time, it has been housed in various local buildings including a community center, library, and hunting club, ranging in distance from a quarter to a half mile from the human medical clinic. At its inception, the veterinary clinic was held in close proximity to the existing health care clinic for people in order to improve cross disciplinary communication including shadowing, interprofessional journal club, and to provide people with collaborative care and convenient, co-located services for their pets. As the veterinary clinic has moved farther from the human medical clinic, connections with the medical team have become more challenging. Furthermore, the COVID-19 pandemic severely hampered this relationship as the provision of health care was interrupted and established connections with student and faculty leaders at the human medical clinic were lost.

In 2021, a relationship with a university-run, Master's Entry Program in Nursing (MEPN) was established to promote interprofessional education and practice, and again collocate health services for animals and people. Pre-licensure nursing students, supervised by registered nurse clinical faculty, attend rounds with veterinary medicine teams and connect with the client while their pet is being seen. The nursing team offers support and advocacy, asking questions about social determinants of health and resource barriers that impact all family members. Nursing students focus on preventive care offering health screenings, including blood glucose and blood pressure checks, as well as health education resources. Donated supplies have periodically allowed for COVID-19 tests and home blood pressure monitors to be provided at no cost to clinic attendees. Nursing students also participate in journal club rounds that occur prior to the clinic and the group debrief at the close of the clinic, prompting discussions on the similarities and differences between human and animal health and healthcare management. This addition of the nursing students realigned the One Health clinic focus by allowing health needs to be addressed for all family members.

### 1.3 Clinic model

The OHC occurs once monthly at a community building on an appointment-based system, accommodating walk-in appointments when possible. Clients are eligible to participate through residency in one of two designated zip codes, though physical proof of residency is not required. Supplies and pharmaceuticals are acquired through a combination of corporate donations, grant funding, and philanthropic support. Financial donations from clients are accepted but not required.

Veterinary and undergraduate students provide the clinic organization with oversight from faculty and staff. At its inception, the faculty veterinarian involvement was on a voluntary basis, but in 2022 funding was secured to provide for a part-time faculty appointment to oversee the program. Additional veterinary oversight during the clinic day is provided by volunteers from both within and outside of the university. The original process to include veterinarians from outside the university involved establishing a faculty appointment without pay for each volunteer. That process took several months to complete and was reported to be a barrier to volunteer veterinarians participating in the clinic. In an effort to encourage a wider volunteer base, the process for enrolling veterinary professionals was modified in 2022 to involve a background check, volunteer application and acknowledgment of our Principles of Community as the only required steps. This process can be completed in < 1 week. Veterinary technician involvement was also initially only available on a volunteer basis, but grant support allowed for the hire of a part-time veterinary technician in 2023. Additional volunteer technicians are recruited in the same fashion as volunteer veterinarians. Nursing clinical instructors are all faculty at the school of nursing and participate as part of their faculty positions.

All veterinary students participate on a volunteer basis and nursing students participate either on a volunteer basis or as part of a class, depending on the time of year. Undergraduate students participate as part of a 1–2 credit course, and the demand for this class far exceeds the available enrollment. The selection process for the undergraduate course includes a comprehensive application that evaluates responses to prompts on topics including Access to Care (12) and One Health (13). Additionally, there is a separate application process to select Spanish language interpreters. To ensure an adequate number of interpreters at each clinic, Spanish speaking undergraduate students who pass a translation competency test are given higher rankings in the course selection process.

The undergraduate students participate in several roles at the clinic depending on their current level of completed training, including medical scribe, veterinary assistant, and Spanish language interpreter. All participating students work in healthcare teams consisting of 1–2 veterinary students, 1 undergraduate student, 1 veterinarian overseeing the team and a nursing student and technician, if available. The nursing faculty circulate during the clinic, providing oversight of their students, as well as direct patient contact.

Recruitment of volunteer students and clinical staff varies seasonally and depending on conflicting activities. Clinic organizers have worked to build a diverse workforce by encouraging students, technicians and veterinarians to bring colleagues. Moreover, grant funding has provided for compensation for volunteer veterinarians and technicians to offset their travel expenses related to clinic attendance.

### 1.4 Clinic data

To describe the current scope of monthly veterinary clinics, veterinary patient records and clinic operational data from July 2022 to July 2024 were reviewed. This time frame was selected to reflect the most current, post-COVID-19 clinic operations. Review of clinic operational data collected and managed by DVM student leadership provided information on the types and numbers of volunteers, vaccinations, medications prescribed, and referral surgeries.

#### 1.4.1 Patient demographics, appointment types, and presenting complaints

**Figure 1:**
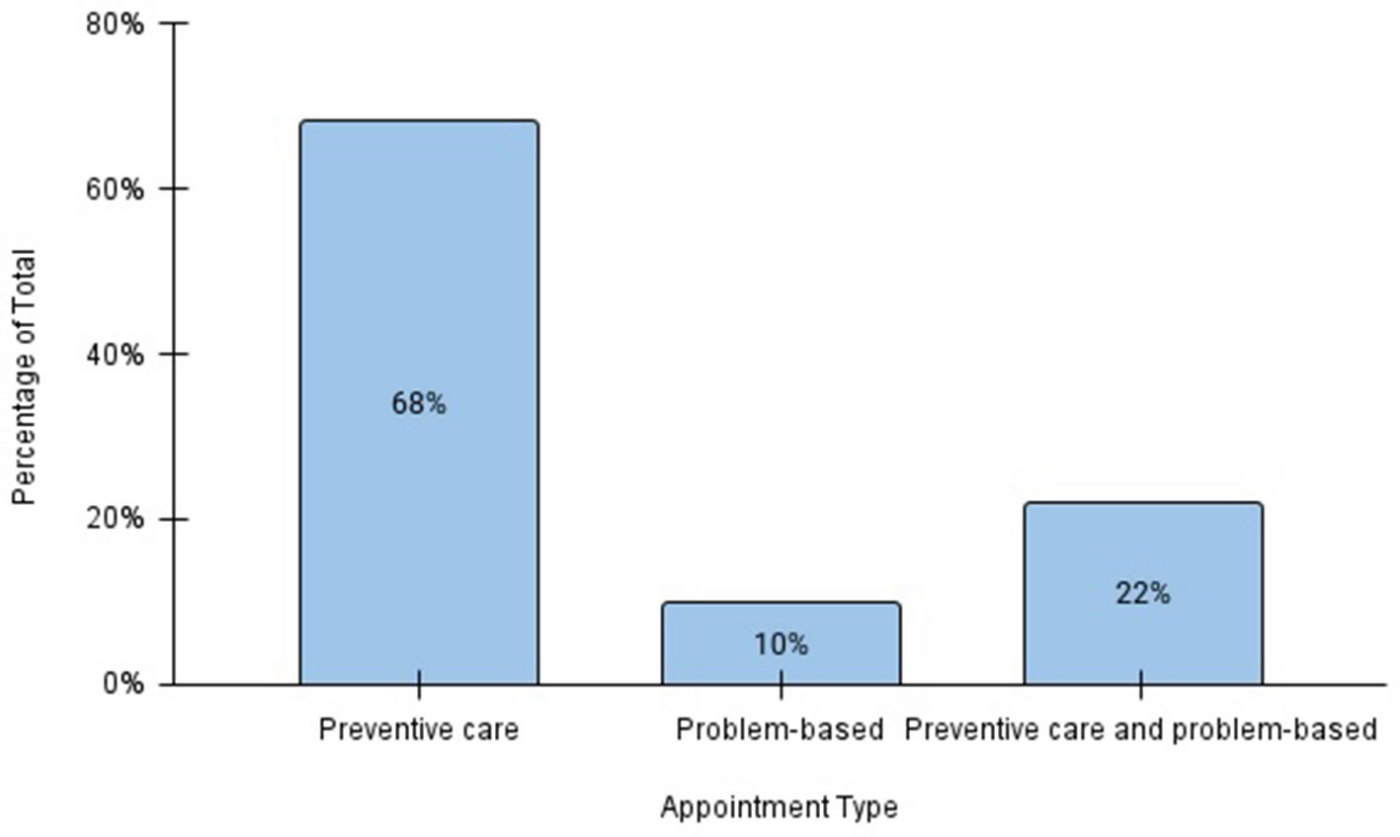
Distribution of veterinary appointment types at the OHC, July 2022–July 2024.

**Figure 2:**
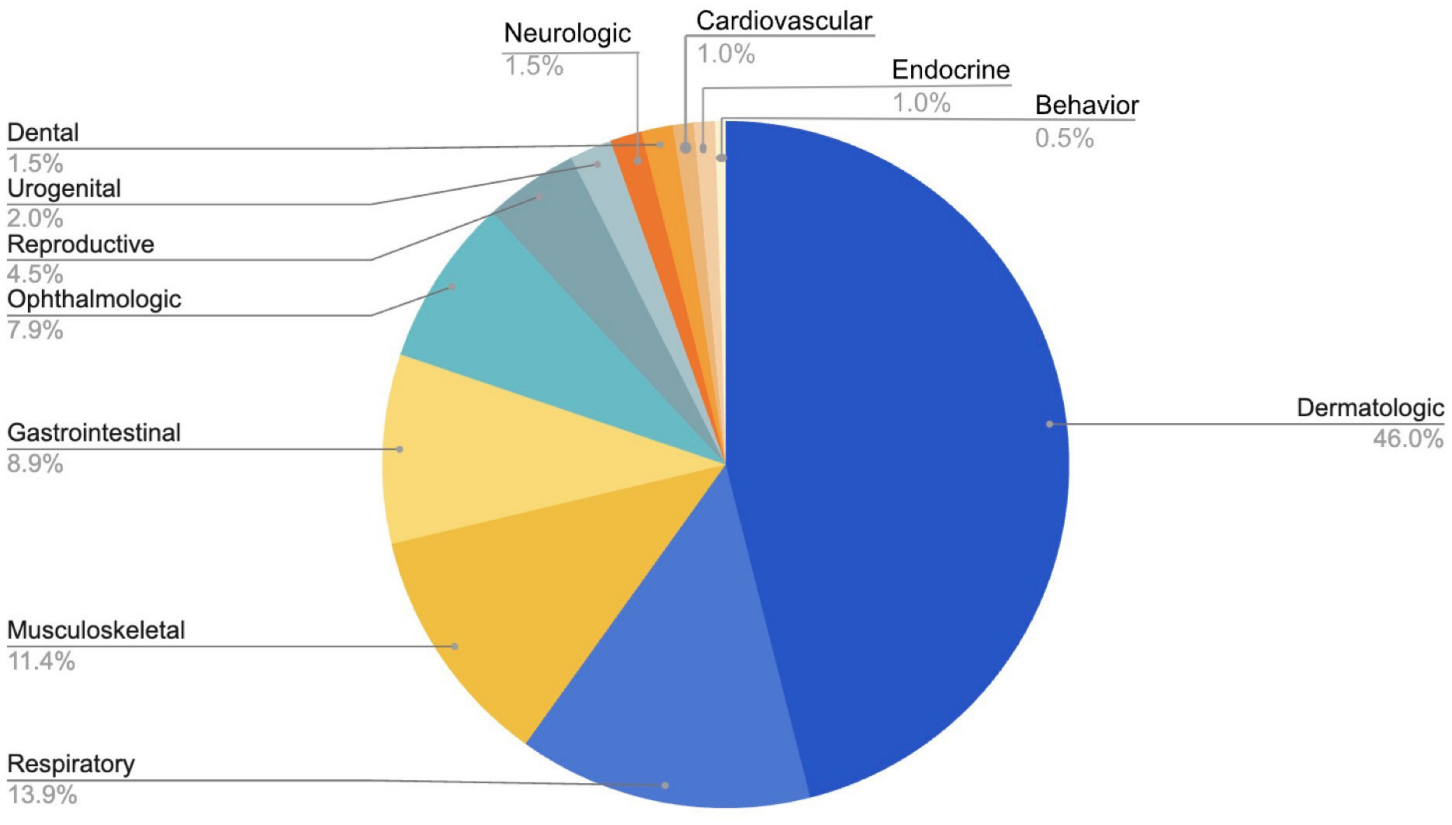
Categories of presenting complaints for veterinary problem-based appointments at the OHC, July 2022–July 2024.

The veterinary clinic provided care for 267 pets during the review period (Table 1). Of these, 72% were dogs and 28% cats. The mean ages of dogs and cats evaluated at the clinic were 5 and 3 years, respectively. Among both species, the majority of animals were sexually intact at initial presentation (63% of dogs and 58% of cats). A total of 618 appointments occurred during the review period, with a mean of 23 visits per clinic day. Preventive care visits were the most common appointment type (68%), followed by combined problem-based and preventive care visits (22%), and purely problem-based visits (10%; Figure 1). Problem-based appointments were offered to those presenting with conditions appropriate for outpatient treatment and if a patient required more intensive management, referral to a full-service veterinary practice was recommended. If patients were stable on examination while presenting for a problem, preventive care was provided during the same visit if indicated. Among problem-based appointments, dermatologic issues were the most common presenting complaints (46%) followed by respiratory (13.9%), musculoskeletal (11.4%), and gastrointestinal (8.9%) concerns (Figure 2).

**Table 1:** Demographics of 267 unique veterinary patients treated at the OHC, July 2022–July 2024.

	**Dog**	**Cat**
Number of patients	193 (72.3%)	74 (27.7%)
**Age**		
Mean and range (years)	5 (0.2–18)	3 (0.2–13)
**Sex**		
Female intact	72 (37.3%)	19 (25.7%)
Female spayed	40 (20.7%)	20 (27.0%)
Male intact	50 (25.9%)	24 (32.4%)
Male neutered	30 (15.5%)	11 (14.9%)

#### 1.4.2 Services provided

In addition to physical examinations and client education, the monthly veterinary clinic provided routine diagnostic testing, vaccinations, endo- and ectoparasite preventives, prescriptions for conditions amenable to outpatient treatment, and referrals for spay, neuter, and mass removals at the teaching hospital. More than half (61%) of the diagnostic procedures performed were canine (vector borne) and feline (retrovirus and heartworm) infectious disease screening tests (Table 2). The remaining 39% of diagnostic procedures performed were ear and fine needle aspirate cytology, ophthalmic tests, and point of care ultrasound. Blood and urine samples collected for complete blood cell count and serum biochemistry panel (9%) and urinalysis (4%) were submitted to clinical diagnostic laboratories for analysis.

**Table 2:** Common veterinary preventative care services provided at the OHC, July 2022–July 2024.

	**Count**	**Percentage (%)**
**Diagnostic tests**		
Canine point of care infectious disease testing	128	44.9
Feline point of care infectious disease testing	47	16.5
Ear cytology	32	11.2
CBC/chemistry panel	26	9.1
FNA	17	6.0
Ophthalmic tests	15	5.3
Urinalysis	11	3.9
Point of care ultrasound	9	3.2
**Surgery referrals**		
Canine ovariohysterectomy	29	38.7
Feline castration	18	24.0
Feline ovariohysterectomy	14	18.7
Canine castration	12	16.0
Other	2	2.7
**Vaccinations**		
*Leptospira* spp.	215	26.2
Rabies	185	22.5
Canine distemper, adenovirus, parainfluenza, parvovirus (DAPP)	180	21.9
Feline leukemia virus (FeLV)	85	10.4
Feline viral rhinotracheitis, calicivirus, panleukopenia (FVRCP)	83	10.1
*Bordetella bronchiseptica*	73	8.9

A total of 821 canine and feline core vaccines, including *Leptospira* spp. and feline leukemia virus vaccines (14–16) were administered with a mean of 33 vaccinations per clinic (Table 2). A total of 75 dogs and cats were referred for surgery. Canine ovariohysterectomy was the most common surgical referral, followed by feline castration, feline ovariohysterectomy, and canine castration (Table 2). There were 2 patients referred for non-preventive care surgeries during the study period for mass removal and laceration repair.

#### 1.4.3 Volunteers

**Figure 3:**
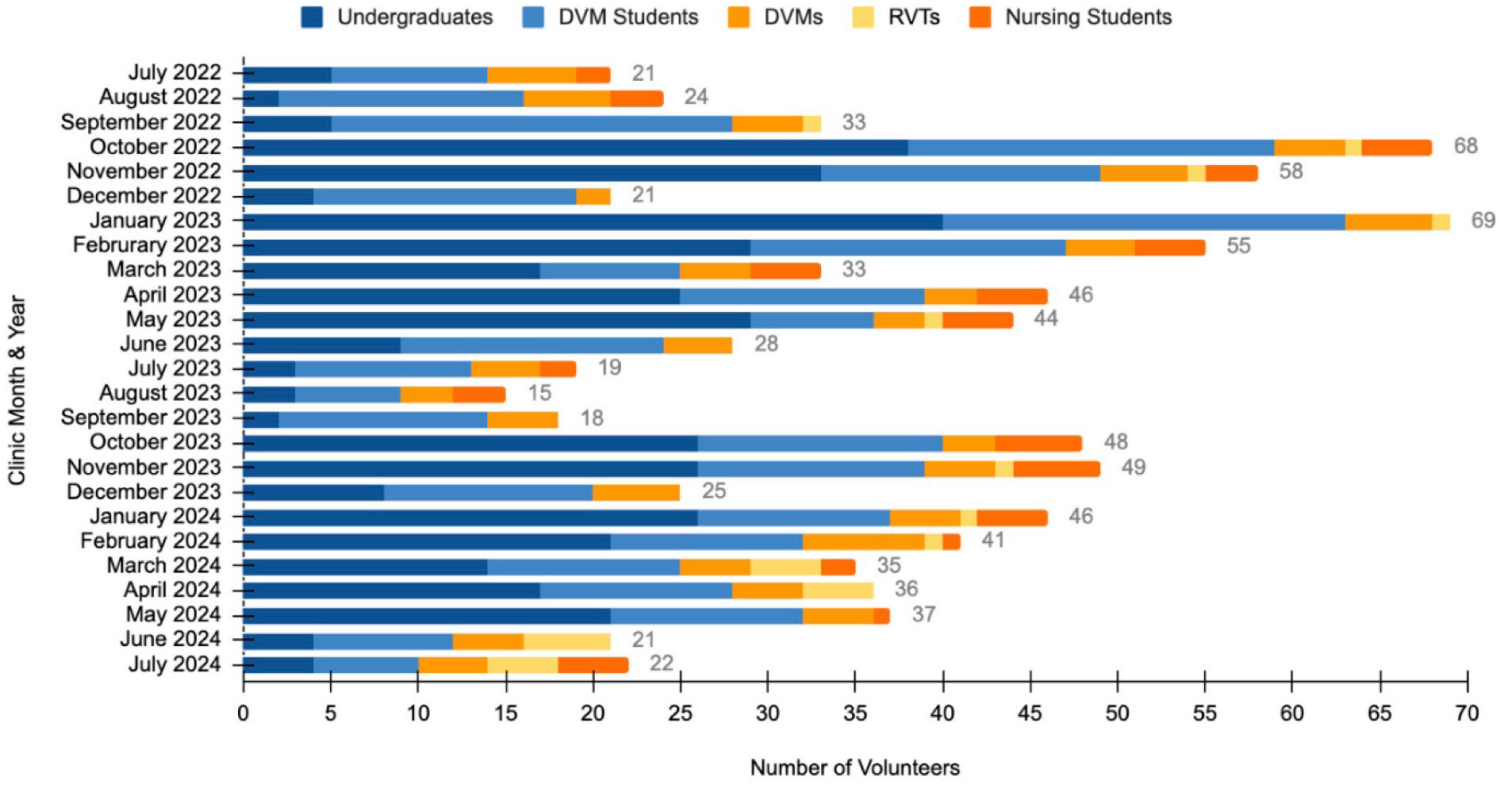
Number and type of volunteers in attendance at the OHC, July 2022–July 2024. The total number of volunteers is represented at the end of each bar.

The total number of volunteers ranged from 15 to 69 per month, with a mean of 36 volunteers per clinic (Figure 3). Students comprised the majority of volunteers with a mean of 12 veterinary students and 16 undergraduate students per clinic. Clinic veterinarian volunteerism varied by month with a mean attendance of 4 per clinic. Similarly, registered veterinary technicians (RVTs) were present at 48% of clinics with a mean of 1 RVT at clinics with technician support. While nursing students were not able to attend all clinic days given scheduling conflicts, when available they ranged from 1 to 5 per clinic with 1–2 clinical faculty.

### 1.5 Challenges and evolution

Internal review of clinic patient and provider safety, student preparedness, and exploration of interprofessional training opportunities was completed by faculty observations done from the spring to fall of 2022. These assessments identified a need for updated student training protocols and increased oversight to achieve an optimal balance of experiential learning and quality patient care. Changes to clinic protocols were implemented from 2022 to 2024. The first tier of educational modifications addressed medical issues as well as patient and provider safety.

At the clinic's inception, veterinary student teams conducted client visits, then presented their findings and recommendations to an attending faculty veterinarian for approval. Veterinary students performed vaccinations and venipuncture as indicated. In these conditions, the supervising veterinarian might oversee 3–5 cases simultaneously. As the caseload grew, supervising veterinarians were responsible for a greater number of patients and their attention became divided, raising concern about the level of oversight affecting student and patient safety, teaching opportunities, and length of visits. In response, the care-team structure was changed to include veterinary students with a range of experience levels, a supervising veterinarian, an undergraduate student, and when available, an RVT and nursing student. This change allowed for a more streamlined approach, direct supervision, and clinical instruction during each patient visit. Working with RVTs familiarizes students with these important members of the veterinary care team, while providing opportunities to learn safe patient handling skills from their wealth of experience. Additionally, online low stress patient handling courses (17) were added to the undergraduate student class to better prepare them for positive patient interactions.

Initially, medications were authorized by faculty veterinarians with students completing all other parts of the process, including requesting the medications from the pharmacy student, and writing prescription instructions. A double-signature system was implemented beginning in November of 2022 to improve prescription accuracy. In current practice, all prescriptions are evaluated and approved by a veterinarian both before and after the medications are filled to ensure accuracy. We also regularly discuss these concepts as a group and provide opportunities for students to ask the clinical instructors prescription-related questions. In addition, as part of their required training, undergraduate students receive a lecture on veterinary and pharmacy law regarding prescription provision, dispensation, and labeling.

The second tier of educational modifications addressed cultural issues and included methods to better prepare students for the unique aspects of the OHC. Programs for underserved (18) communities do not always provide students with training on the causes of health disparities (19) but those that offer information about the culture and community result in increased opportunities to actualize cultural humility appreciation and understanding (20–23). The undergraduate course curriculum was updated and includes new information on the principles of ethical community engagement (24) cultural humility (25, 26), and the social determinants of health (27–32). For the veterinary student volunteers, a video that highlights our philosophy of ethical community engagement was created and is a prerequisite to clinic attendance.

Each clinic day begins with a pre-clinic meeting of all students and clinical instructors to review clinic flow, announce healthcare teams, and share pertinent information which might include seasonal or weather-related reminders. Core principles are reviewed: translation etiquette, how to embrace spectrum of care (33–35) and contextualized care (36–38), and the importance of implementing relationship-centered communication and shared-decision making (26, 39, 40). While the scope of these topics exceeds the time available in the pre-clinic meeting, the concepts are introduced, and students are encouraged to work with the clinical instructors throughout the clinic day to discuss further.

An important service offered at this OHC is interpretation/translation services, given that a large proportion of clients speak Spanish as their primary language. Language barriers not only impede access to veterinary care (41), but there is also strong evidence indicating that they can lead to health consequences for human patients, such as prolonged hospital stays and development of serious medical conditions (42–45). Previous studies have shown that patients prefer to receive care from providers who speak their primary language (46, 47) and that if interpreter services are needed, students should be trained in proper interpretation etiquette (48, 49). Interpretation services at this OHC are provided by professional and student volunteers who have professional Spanish proficiency. To improve student preparedness in this area and facilitate best patient outcomes, all professional and student volunteers at the clinic are provided with a brief training and modeling on how to properly communicate with clients via an interpreter. Undergraduate volunteers also receive a lecture on interpretation and translation etiquette, and may take on the role of interpreter after passing a translation proficiency exam. Students receive real-time feedback during the clinic on how to best utilize an interpreter to facilitate open conversations with clients who would otherwise not receive care due to their language barrier.

The third tier of modifications included increased opportunities for education about clinical One Health and time for self-reflection. Interprofessional One Health journal rounds had previously been part of the OHC but were discontinued in 2020 as a result of the COVID-19 pandemic. The new partnership with the school of nursing allowed for reintroduction of One Health rounds during the pre-clinic meeting. Nursing and veterinary student leaders are invited to select and lead the discussion around a One Health related journal article with faculty or staff support.

A group debrief is held at the end of each clinic to facilitate deeper discussion of collaborative case management in a community OHC. The value of service learning and One Health education is well documented in the literature (50–52) and a community One Health clinic adds an opportunity that is rarely available in a tertiary care center setting. During this session, the teams gather to discuss similarities and differences in our professions' goals, priorities, and methods to achieve positive patient outcomes. Additionally, students are challenged to consider the impact of collaboration in breaking down barriers faced by bonded families seeking care. Faculty and staff model and implement interprofessional collaboration, which has been shown to have documented benefits in both medical care delivery (53–55) and education (56–58). The students are encouraged to actively participate and delve deeper into subjects, including discussions on how the social determinants of health can lead to health disparities and how our professions may be more effective if we work together to reduce barriers to care. Reflections gathered from the undergraduate students who participated in the course and clinic demonstrate the effectiveness of the aforementioned strategies:

“I want to continue to be a part of [the OHC] not only to foster my clinical skills, but more significantly to serve the [rural northern California] community through ethical engagement. … I learned different factors such as cultural barriers, financial barriers, etc., and how a healthcare professional can change how they behave/interact so that we can be on the same page as the people we are serving.”

“Engaging with the [rural northern California] community and attending [the undergraduate course] lectures have broadened my perspective on healthcare accessibility, strengthening my empathy and commitment to providing compassionate, inclusive veterinary services. …[The OHC] has shaped my vision in veterinary medicine, and I am eager to keep growing and serving in this capacity.”

“…I've learned why One Health is a crucial and effective approach to healthcare, and the importance of contextualized care that prioritizes the client's needs and circumstances over a ‘gold standard'. [The OHC undergraduate class] strengthened my interpersonal skills, teaching me to communicate effectively and empathetically in a clinical setting so clients feel validated and supported.”

## 2 Discussion

The OHC described reduces healthcare barriers for people and pets while providing a range of training opportunities for students that complement clinical experience gained in a tertiary care institution when there is a balance of student autonomy and clinical instruction. A current challenge in veterinary education is that the training in many veterinary colleges is largely focused on sophisticated procedures, resulting in veterinarians lacking fundamental knowledge, skills, and comfort on how to offer care across a spectrum (33, 34). Additionally, most veterinary training programs are within tertiary care institutions where the “gold standard” (59) of care is taught and defined as the most technically advanced and often most invasive option. This discrepancy between what is taught in veterinary teaching programs and what can be done when clients face barriers to care results in conflict and moral distress for the provider (60). Recent literature also suggests that veterinary education should move away from the original definition of “gold standard” (37, 61, 62) and instead train veterinary professionals to use spectrum of care as a tool to help deliver contextualized care, working with clients to select a diagnostic and treatment plan that matches the needs of the unique client-patient pair. In addition, the American Association of Veterinary Medical Colleges has recently outlined new competencies around spectrum of care (63). The OHC offers an opportunity to model and train spectrum of care and contextualized care while helping provide healthcare opportunities to an underserved community.

New veterinary school graduates have also expressed a desire to gain increased experience in general practice (64) and point of care testing (65). The scope of care at the OHC includes administration of core vaccinations, prescription of parasite preventatives, and the management commonly reported presenting complaints such as dermatologic issues, respiratory symptoms, musculoskeletal pain, and gastrointestinal signs (Table 2, Figure 2). The students also utilize point of care testing to screen for common infectious diseases, perform routine skin, ear, and eye testing, and use point of care ultrasound, all of which align with skills needed in primary care practice [(66); Table 2].

The community OHC model offers an additional excellent teaching opportunity for veterinary students to develop and practice skills necessary to the safe and legal prescription of medical therapy that they will use in a practice setting. Unlike human medical systems, private and corporate veterinary hospitals rarely have a pharmacist on staff. Safety and accuracy of prescriptions is a legal requirement, and studies have shown that prescription errors can result in serious issues for both the patient and the provider (67, 68). The OHC offers a balance of student autonomy and oversight in pharmacy management that is frequently unavailable in a tertiary care teaching institution.

Despite the robust caseload and array of clinical training opportunities available at the OHC, one of the major limitations of the current model is that participation is largely limited to veterinary students in the pre-clinical years. Students in the clinical year are rarely available during the OHC days largely due to their obligations to other clinical rotations. It is unknown to what extent participation in the OHC as a pre-clinical student translates into confidence and competence in a final year veterinary student, however opportunities for students in their clinical year at this veterinary school to participate in formal, non-externship clinical rotations outside of a tertiary referral center are limited. Future goals are to develop a formal fourth year rotation in the OHC or similar clinic, as well as to formally investigate the role of student participation in teaching cultural humility.

The new partnership with the school of nursing, reinitiation of the One Health journal discussion, and the addition of the post-clinic debriefs augment the IPE opportunities at the OHC. These new aspects encourage students to self-reflect, follow the principles of ethical community engagement, and consider how the social determinants of health affect health outcomes for people and pets (69), while allowing participants to take actionable steps toward improving care. It has been shown that access to veterinary care is a One Health issue (70). Clinical IPE opportunities can increase knowledge about the application of One Health practices (58, 71), improve competence and confidence in teamwork skills (72), while minimizing barriers to healthcare for people and their pets, thus taking these concepts from theoretical to practical. The veterinary partnership with this community and the human nursing team also aligns with core principles of the American Animal Hospital Association Community Care Guidelines for Small Animal Practice, a recently developed benchmark for best practices in small animal medicine which outlines the importance of family-centered healthcare (73).

There are numerous communities in the United States and other countries that lack adequate medical or veterinary healthcare facilities and personnel, and studies have found a relationship between health professional students' exposure to rural and underserved care and their future practice choices (74, 75). The One Health clinic model could serve as a tool to improve access to care in both rural and urban settings. Furthermore, IPE in a community OHC may improve connections for veterinarians and nurses in these geographic regions and demographics (76, 77).

The collaboration of various professions and student groups required to run a OHC has numerous benefits but also comes with associated challenges. These included physical site limitations, coordination of undergraduate and professional school schedules, and recruitment of clinical instructors with the time and dedication to provide appropriate training and supervision for a community-based SRFC. Recruitment of volunteer veterinarians and technicians was improved by instituting policies to reduce barriers to participation as well as offering compensation for travel to the clinic. Further avenues that could be explored include offering veterinarians and technicians continuing education credits for service during the clinic when coupled with supplemental training videos, much like has been accomplished with high-quality high-volume spay-neuter training clinics (78, 79). Long term strategies for sustainability of funding could include a hybrid structure of university support for teaching; corporate, private donor and grant support of products; and the consideration of a small co-pay by clients of the OHC.

Participation in student-run clinics is affected by university calendar systems, class size, and policies. Undergraduate students and veterinary students participated in every clinic, but there were consistently low volunteer numbers attending clinics in the months of June, July, August, September, and December, which correspond to summer and winter university breaks. As visible in Figure 3, there was also a restructuring of the undergraduate class for the 2023–2024 academic year that decreased the number of volunteer spots available. This reduction was aimed as a method to reduce crowding and stress during patient visits. Participation of nursing students varied throughout the year based on their academic calendar and if their active enrollment overlapped with the scheduled clinics. The OHC participation was integrated into a nursing course in 2024 which allowed for the same four students to attend three consecutive clinics. Clinical instructors of both nursing and veterinary medicine noted increased engagement of clients with consistent nursing faculty and student participation. Greater consistency facilitated follow-up conversations about management of human health issues such as hypertension and diabetes mellitus among other topics. Incorporating the OHC into the curriculum for students of nursing and veterinary medicine would create improved continuity of care and could be beneficial for students, clients, and patients (80).

In conclusion, evaluation of the scope of practice, challenges, and evolution of a student-run, free OHC demonstrates this model of service-learning experience provides preventative healthcare options for people and pets experiencing barriers to care, while offering student training opportunities that complement current veterinary educational systems. Further study is indicated to measure the long-term health benefits on the people and pets served at the OHC, and if the students of both disciplines demonstrate improved practice readiness, less moral distress, and greater interprofessional collaboration skills upon graduation.

## Data Availability

The original contributions presented in the study are included in the article/supplementary material, further inquiries can be directed to the corresponding author/s.

## References

[1] Jordan T Lem M. One Health, One Welfare: education in practice veterinary students' experiences with Community Veterinary Outreach. Can Vet J. (2014) 55:1203–6. 25477552 PMC4231813

[2] Powell L Walsh M Reinhard CL Jankowski K Watson B. One Health clinic promotes veterinarian-client trust among underserved pet owners and provides learning opportunities for veterinary students. J Am Vet Med Assoc. (2022) 260:931–9. 10.2460/javma.21.06.0274 35298404

[3] Society of Student-Run Free Clinics. Available online at: https://www.studentrunfreeclinics.org/ (accessed March 6, 2025).

[4] Rivkin-Fish M. Learning the moral economy of commodified health care: “community education,” failed consumers, and the shaping of ethical clinician-citizens. Cult Med Psychiatry. (2011) 35:183–208. 10.1007/s11013-011-9208-0 21560031

[5] Vinarcsik L Wilson Y. Beyond good intentions: student run free clinics as a reflection of a broken system. Am J Bioeth. (2022) 22:27–9. 10.1080/15265161.2022.2027567 35258433

[6] Peoples N Gebert JT Clark D. Turning good intentions into good outcomes: ethical dilemmas at a student-run clinic and a rubric for reflective action. Med Humanit. (2024) 50:179–84. 10.1136/medhum-2023-012695 37696600

[7] Access to Veterinary Care Coalition. Access to Veterinary Care: Barriers, Current Practices and Public Policy. Knoxville, TN: U. of Tennessee (2018).34242249

[8] Knights Landing, California Population (2024). Available online at: https://worldpopulationreview.com/us-cities/california/knights-landing (accessed February 21, 2025).

[9] Pet Policy—Yolobus. Available online at: https://yolobus.com/pet-policy/ (accessed March 13, 2025).

[10] Sweeney JM Zielinska Crook P Deeb-Sossa N Tu B Dear JD Mazet JAK. Clinical one health: a novel healthcare solution for underserved communities. One *Health*. (2018) 6:34–6. 10.1016/j.onehlt.2018.10.003 30386814 PMC6205339

[11] Courtenay M Sweeney J Zielinska P Brown Blake S La Ragione R. One Health: an opportunity for an interprofessional approach to healthcare. J Interprof Care. (2015) 29:641–2. 10.3109/13561820.2015.1041584 26652637

[12] Pasteur K Diana A Yatcilla JK Barnard S Croney CC. Access to veterinary care: evaluating working definitions, barriers, and implications for animal welfare. Front Vet Sci. (2024) 11:1335410. 10.3389/fvets.2024.1335410 38304544 PMC10830634

[13] One Health High-Level Expert Panel (OHHLEP) Adisasmito WB Almuhairi S Behravesh CB Bilivogui P Bukachi SA . One Health: a new definition for a sustainable and healthy future. PLoS Pathog. (2022) 18:e1010537. 10.1371/journal.ppat.1010537 35737670 PMC9223325

[14] Stone AE Brummet GO Carozza EM Kass PH Petersen EP Sykes J. 2020 AAHA/AAFP feline vaccination guidelines. J Feline Med Surg. (2020) 22:813–30. 10.1177/1098612X20941784 32845224 PMC11135662

[15] Ellis J Marziani E Aziz C Brown CM Cohn LA Lea C. 2022 AAHA canine vaccination guidelines (2024 update). J Am Anim Hosp Assoc. (2024) 60:1–19. 10.5326/JAAHA-MS-7468 39480742

[16] Sykes JE Francey T Schuller S Stoddard RA Cowgill LD Moore GE. Updated ACVIM consensus statement on leptospirosis in dogs. J Vet Intern Med. (2023) 37:1966–82. 10.1111/jvim.16903 37861061 PMC10658540

[17] Fear Free Pets—Taking the “Pet” Out of “Petrified” for All Animals. Available online at: https://fearfreepets.com/ (accessed December 20, 2024).

[18] Roberts C Woodsworth J Carlson K Reeves T Epp T. Defining the term “underserved:” a scoping review towards a standardized description of inadequate access to veterinary services. Can Vet J. (2023) 64:941–50. 37780475 PMC10506354

[19] Like RC. Educating clinicians about cultural competence and disparities in health and health care. J Contin Educ Health Prof. (2011) 31:196–206. 10.1002/chp.20127 21953661

[20] Allen J. Improving cross-cultural care and antiracism in nursing education: a literature review. Nurse Educ Today. (2010) 30:314–20. 10.1016/j.nedt.2009.08.007 19758731

[21] Alvarez EE Gilles WK Lygo-Baker S Chun R. Teaching cultural humility and implicit bias to veterinary medical students: a review and recommendation for best practices. J Vet Med Educ. (2020) 47:2–7. 10.3138/jvme.1117-173r1 30920944

[22] Alvarez EE Gilles WK Lygo-Baker S Howlett B Chun R. How to approach cultural humility debriefing within clinical veterinary environments. J Vet Med Educ. (2021) 48:256–62. 10.3138/jvme.2019-0039 32412367

[23] Tsimaras T Wallace JE Adams C Baker TM Kutz SJ. Actualizing cultural humility: an exploratory study of veterinary students' participation in a northern community health rotation. J Vet Med Educ. (2023) 50:205–16. 10.3138/jvme-2021-0130 35385371

[24] Watson B Berliner E DeTar L McCobb E Frahm-Gilles W Henry E. Principles of veterinary community engagement. JSMCAH. (2024) 3:2024. 10.56771/VCEprinciples.2024

[25] Tervalon M Murray-García J. Cultural humility versus cultural competence: a critical distinction in defining physician training outcomes in multicultural education. J Health Care Poor Underserved. (1998) 9:117–25. 10.1353/hpu.2010.0233 10073197

[26] Englar RE Graham Brett T. Integrating communication skills, awareness of self and others, and reflective feedback into one inclusive anatomical representation of relationship-centered health care. J Vet Med Educ. (2023) 50:399–412. 10.3138/jvme-2022-0060 36538494

[27] Social Determinants of Health (SDOH) | About CDC | CDC. Available online at: https://www.cdc.gov/about/priorities/why-is-addressing-sdoh-important.html (accessed December 10, 2024).

[28] Card C Epp T Lem M. Exploring the social determinants of animal health. J Vet Med Educ. (2018) 45:437–47. 10.3138/jvme.0317-047r 30285599

[29] Shih HY Paterson MBA Phillips CJC. Socioeconomic influences on reports of canine welfare concerns to the royal society for the prevention of cruelty to animals (RSPCA) in queensland, australia. Animals. (2019) 9:e100711. 10.3390/ani9100711 31547537 PMC6827051

[30] Dyer JL Milot L. Social vulnerability assessment of dog intake location data as a planning tool for community health program development: a case study in Athens-Clarke County, GA, 2014-2016. PLoS ONE. (2019) 14:e0225282. 10.1371/journal.pone.0225282 31790438 PMC6886854

[31] Spencer T Behar-Horenstein L Aufmuth J Hardt N Applebaum JW Emanuel A. Factors that influence intake to one municipal animal control facility in Florida: a qualitative study. Animals. (2017) 7:e70048. 10.3390/ani7070048 28665336 PMC5532563

[32] McDowall S Hazel S Learey T Stokes T McArthur M. Exploring social determinants of health in veterinary technology: a workshop approach to enhancing companion animal welfare and student awareness. J Vet Med Educ. (2025) 52:e20240157. 10.3138/jvme-2024-0157 40072319

[33] Stull JW Shelby JA Bonnett BN Block G Budsberg SC Dean RS. Barriers and next steps to providing a spectrum of effective health care to companion animals. J Am Vet Med Assoc. (2018) 253:1386–9. 10.2460/javma.253.11.1386 30451620

[34] Fingland RB Stone LR Read EK Moore RM. Preparing veterinary students for excellence in general practice: building confidence and competence by focusing on spectrum of care. J Am Vet Med Assoc. (2021) 259:463–70. 10.2460/javma.259.5.463 34388008

[35] Brown CR Garrett LD Gilles WK Houlihan KE McCobb E Pailler S. Spectrum of care: more than treatment options. J Am Vet Med Assoc. (2021) 259:712–7. 10.2460/javma.259.7.712 34516261

[36] Weiner SJ. Contextualizing care: an essential and measurable clinical competency. Patient Educ Couns. (2022) 105:594–8. 10.1016/j.pec.2021.06.016 34158194

[37] Skipper A Gray C Serlin R O'Neill D Elwood C Davidson J. “Gold standard care” is an unhelpful term. Vet Rec. (2021) 189:331. 10.1002/vetr.1113 34677842

[38] Skipper A O'Neill D Serlin R Davidson J Elwood C Gray C. Contextualised care: faddish or foundational? Vet Rec. (2024) 195:117. 10.1002/vetr.4567 38757851

[39] Hirschmann K Rosler G Fortin Vi AH. “For me, this has been transforming”: a qualitative analysis of interprofessional relationship-centered communication skills training. J Patient Exp. (2020) 7:1007–14. 10.1177/2374373520962921 33457539 PMC7786664

[40] Janke N Shaw JR Coe JB. On-site communication skills education increases appointment-specific client satisfaction in four companion animal practices in Texas. J Am Vet Med Assoc. (2022) 260:1711–20. 10.2460/javma.22.06.0242 35976983

[41] Hoffman CL Spencer TG Makolinski KV. Assessing the impact of a virtual shelter medicine rotation on veterinary students' knowledge, skills, and attitudes regarding access to veterinary care. Front Vet Sci. (2021) 8:783233. 10.3389/fvets.2021.783233 34977216 PMC8716626

[42] Cohen AL Rivara F Marcuse EK McPhillips H Davis R. Are language barriers associated with serious medical events in hospitalized pediatric patients? Pediatrics. (2005) 116:575–9. 10.1542/peds.2005-0521 16140695

[43] Flores G Tomany-Korman SC. The language spoken at home and disparities in medical and dental health, access to care, and use of services in US children. Pediatrics. (2008) 121:e1703–14. 10.1542/peds.2007-2906 18519474

[44] Lindholm M Hargraves JL Ferguson WJ Reed G. Professional language interpretation and inpatient length of stay and readmission rates. J Gen Intern Med. (2012) 27:1294–9. 10.1007/s11606-012-2041-5 22528618 PMC3445680

[45] Levas MN Dayan PS Mittal MK Stevenson MD Bachur RG Dudley NC. Effect of Hispanic ethnicity and language barriers on appendiceal perforation rates and imaging in children. J Pediatr. (2014) 164:1286–91.e2. 10.1016/j.jpeds.2014.01.006 24565425

[46] Ferguson WJ Candib LM. Culture, language, and the doctor-patient relationship. Fam Med. (2002) 34:353–61. 12038717

[47] Zamudio CD Sanchez G Altschuler A Grant RW. Influence of language and culture in the primary care of Spanish-speaking Latino adults with poorly controlled diabetes: a qualitative study. Ethn Dis. (2017) 27:379–86. 10.18865/ed.27.4.379 29225438 PMC5720947

[48] Vargas Pelaez AF Ramirez SI Valdes Sanchez C Piedra Abusharar S Romeu JC Carmichael C. Implementing a medical student interpreter training program as a strategy to developing humanism. BMC Med Educ. (2018) 18:141. 10.1186/s12909-018-1254-7 29914460 PMC6006684

[49] Nguyen Q Flora J Basaviah P Bryant M Hosamani P Westphal J. Interpreter and limited-English proficiency patient training helps develop medical and physician assistant students' cross-cultural communication skills. BMC Med Educ. (2024) 24:185. 10.1186/s12909-024-05173-z 38395858 PMC10893691

[50] NAVMEC Board of Directors. The North American veterinary medical education consortium (NAVMEC) looks to veterinary medical education for the future: “roadmap for veterinary medical education in the 21st century: responsive, collaborative, flexible.” *J Vet Med Educ*. (2011) 38:320–7. 10.3138/jvme.38.4.320

[51] King E Mueller M Wolfus G McCobb E. Assessing service-learning in community-based veterinary medicine as a pedagogical approach to promoting student confidence in addressing access to veterinary care. Front Vet Sci. (2021) 8:644556. 10.3389/fvets.2021.644556 34222392 PMC8245678

[52] Tan J-Y Poitras Pratt Y Danyluk P. “First, do no harm”: systematic program evaluation of an equine veterinary service-learning initiative with Indigenous communities in Canada. BMC Med Educ. (2024) 24:287. 10.1186/s12909-024-05234-3 38486267 PMC10941546

[53] Gaboury I Bujold M Boon H Moher D. Interprofessional collaboration within Canadian integrative healthcare clinics: key components. Soc Sci Med. (2009) 69:707–15. 10.1016/j.socscimed.2009.05.048 19608320

[54] Karam M Brault I Van Durme T Macq J. Comparing interprofessional and interorganizational collaboration in healthcare: a systematic review of the qualitative research. Int J Nurs Stud. (2018) 79:70–83. 10.1016/j.ijnurstu.2017.11.002 29202313

[55] MacLeod MLP Hanlon N Reay T Snadden D Ulrich C. Partnering for change. J Health Organ Manag. (2019) 34:255–72. 10.1108/JHOM-02-2019-0032 31854955 PMC7410305

[56] Sick B Sheldon L Ajer K Wang Q Zhang L. The student-run free clinic: an ideal site to teach interprofessional education? J Interprof Care. (2014) 28:413–8. 10.3109/13561820.2014.907779 24749742

[57] Lie DA Forest CP Walsh A Banzali Y Lohenry K. What and how do students learn in an interprofessional student-run clinic? An educational framework for team-based care. Med Educ Online. (2016) 21:31900. 10.3402/meo.v21.31900 27499364 PMC4976304

[58] Estrada AH Samper J Stefanou C Blue A. Contemporary challenges for veterinary medical education: examining the state of inter-professional education in veterinary medicine. J Vet Med Educ. (2022) 49:71–9. 10.3138/jvme-2020-0066 33661086

[59] Block G. A new look at standard of care. J Am Vet Med Assoc. (2018) 252:1343–4. 10.2460/javma.252.11.1343 29772971

[60] Moses L Malowney MJ Wesley Boyd J. Ethical conflict and moral distress in veterinary practice: a survey of North American veterinarians. J Vet Intern Med. (2018) 32:2115–22. 10.1111/jvim.15315 30320478 PMC6271308

[61] Englar RE. Recasting the gold standard—part I of II: delineating healthcare options across a continuum of care. J Feline Med Surg. (2023) 25:1098612X231209855. 10.1177/1098612X231209855 38131211 PMC10811762

[62] Englar RE. Recasting the gold standard—part II of II: communicating healthcare options along a continuum of care. J Feline Med Surg. (2023) 25:1098612X231215639. 10.1177/1098612X231215639 38131202 PMC10811761

[63] The Spectrum of Care Initiative—AAVMC. Available online at: https://www.aavmc.org/the-spectrum-of-care-initiative/ (accessed March 12, 2025).

[64] Dixon WHR Kinnison T May SA. Understanding the primary care paradigm: an experiential learning focus of the early veterinary graduate. Vet Rec. (2017) 181:480. 10.1136/vr.104268 28893973

[65] Roth IG Meindl AG Eckman SL Franklin AL. Eliciting the student perspective on point-of-care diagnostic testing in association with a primary care rotation. J Vet Med Educ. (2019) 46:225–34. 10.3138/jvme.0817-102r1 31120411

[66] Robinson NJ Dean RS Cobb M Brennan ML. Investigating common clinical presentations in first opinion small animal consultations using direct observation. Vet Rec. (2015) 176:463. 10.1136/vr.102751 25564472 PMC4431344

[67] Khalil H Bell B Chambers H Sheikh A Avery AJ. Professional, structural and organisational interventions in primary care for reducing medication errors. Cochrane Database Syst Rev. (2017) 10:CD003942. 10.1002/14651858.CD003942.pub3 28977687 PMC6485628

[68] Shalviri G Mohebbi N Mirbaha F Majdzadeh R Yazdizadeh B Gholami K. Improving adverse drug event reporting by healthcare professionals. Cochrane Database Syst Rev. (2024) 10:CD012594. 10.1002/14651858.CD012594.pub2 39470185 PMC11520514

[69] McDowall S Hazel SJ Chittleborough C Hamilton-Bruce A Stuckey R Howell TJ. The impact of the social determinants of human health on companion animal welfare. Animals. (2023) 13:1113. 10.3390/ani13061113 36978653 PMC10044303

[70] Blackwell MJ O'Reilly A. Access to veterinary care–a national family crisis and case for one health. Adv Small Anim Care. (2023) 4:145–57. 10.1016/j.yasa.2023.05.003

[71] Tucker C Keyel J Blue A Chun R Estrada A Khalili H. The intersection of Interprofessional Education and One Health: a qualitative study in human and veterinary medical institutions. One *Health*. (2024) 19:100767. 10.1016/j.onehlt.2024.100767 39113902 PMC11305270

[72] Williams KN Lazzara EH Hernandez J Klocko D Chandran N Paquette SL. Integrating competency-based, interprofessional teamwork education for students: guiding principles to support current needs and future directions. Front Med. (2024) 11:1490282. 10.3389/fmed.2024.1490282 39839636 PMC11748182

[73] Greenberg M McCants D Alvarez E Berliner E Blackwell M McCobb E. 2024 AAHA community care guidelines for small animal practice. J Am Anim Hosp Assoc. (2024) 60:227–46. 10.5326/JAAHA-MS-7464 39480743

[74] Noya F Carr S Freeman K Thompson S Clifford R Playford D. Strategies to facilitate improved recruitment, development, and retention of the rural and remote medical workforce: a scoping review. Int J Health Policy Manag. (2022) 11:2022–37. 10.34172/ijhpm.2021.160 34973053 PMC9808272

[75] Leaune E Rey-Cadilhac V Oufker S Grot S Strowd R Rode G. Medical students attitudes toward and intention to work with the underserved: a systematic review and meta-analysis. BMC Med Educ. (2021) 21:129. 10.1186/s12909-021-02517-x 33627102 PMC7905612

[76] Wolfgang R Wakely L Smith T Burrows J Little A Brown LJ. Immersive placement experiences promote rural intent in allied health students of urban and rural origin. J Multidiscip Healthc. (2019) 12:699–710. 10.2147/JMDH.S214120 31692520 PMC6711721

[77] Berrada M Raboisson D Lhermie G. Effectiveness of rural internships for veterinary students to combat veterinary workforce shortages in rural areas. PLoS ONE. (2024) 19:e0294651. 10.1371/journal.pone.0294651 38451938 PMC10919651

[78] ASPCA Spay/Neuter Alliance Veterinarian Training Program | ASPCApro. Available online at: https://www.aspcapro.org/training-aspca-vet-training/aspca-spayneuter-alliance-veterinarian-training-program (accessed February 25, 2025).

[79] Vet Training Project | Camp LA. Available online at: https://www.campla.org/vet-training-project (accessed February 21, 2025).

[80] Alvarez EE Schultz K Lygo-Baker S Chun R. Veterinary student skills learned at an access to care clinic: beyond medicine and surgery. J Vet Med Educ. (2024) 52:e20240034. 10.3138/jvme-2024-0034 39699996

